# An Overview of the Role of Innate Lymphoid Cells in Gut Infections and Inflammation

**DOI:** 10.1155/2014/235460

**Published:** 2014-07-01

**Authors:** Silvia Sedda, Irene Marafini, Michele M. Figliuzzi, Francesco Pallone, Giovanni Monteleone

**Affiliations:** Department of Systems Medicine, University of Rome “Tor Vergata”, 00133 Rome, Italy

## Abstract

Innate lymphoid cells (ILCs) are a group of hematopoietic cells devoid of antigen receptors that have important functions in lymphoid organogenesis, in the defense against extracellular pathogens, and in the maintenance of the epithelial barrier. Three distinct groups of ILCs have been identified on the basis of phenotypic and functional criteria and termed ILCs1, ILCs2, and ILCs3. Specifically, ILCs1 express the transcription factor T-bet and secrete T helper type-1- (Th1-) related cytokines, ILCs2 are dependent on the transcription factor ROR*α* and express Gata-3 and the chemokine receptor homologous molecule (CRTH2) and produce Th2-related cytokines, and ILCs3 express the transcription factor ROR*γ*t and synthesize interleukin- (IL-) 17, IL-22, and, under specific stimuli, interferon-*γ*. ILCs represent a relatively small population in the gut, but accumulating evidence suggests that these cells could play a decisive role in orchestrating both protective and detrimental immune responses. In this review, we will summarize the present knowledge on the distribution of ILCs in the intestinal mucosa, with particular focus on their role in the control of both infections and effector cytokine response in immune-mediated pathologies.

## 1. Introduction

**Figure 1:**
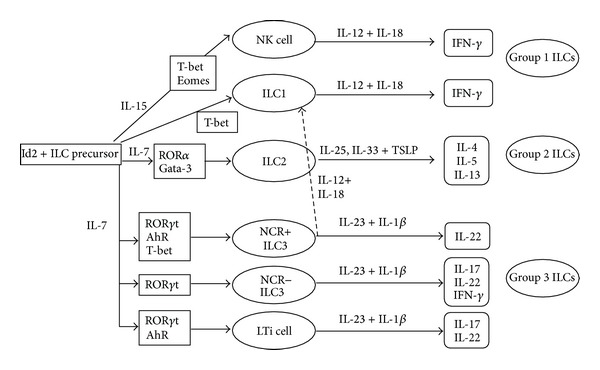
Schematic view of the differentiation of various innate lymphoid cells (ILCs), of the factors involved in such a differentiation, and of the cytokines produced by distinct subsets of ILCs. Id2, inhibitor of DNA binding-2; Gata-3, Gata-binding protein 3; ROR*α*, retinoic acid receptor-related orphan receptor-*α*; Eomes, eomesodermin; NK, natural killer; LTi, lymphoid tissue-inducer; IL, interleukin; NCR, natural cytotoxicity triggering receptor; IFN-*γ*, interferon-*γ*; AhR, aryl hydrocarbon receptor; TSLP, thymic stromal lymphopoietin.

Cells of the innate immune system (i.e., dendritic cells, macrophages, NK cells, and neutrophils) have the ability to recognize and rapidly respond to pathogens with production of various cytokines, which in turn regulate the antigen-driven differentiation of cells of the adaptive immune system (i.e., T and B lymphocytes). This process triggers an effective and specific response, which eliminates pathogens and resolves inflammation and tissue damage [1]. Effector cytokines are also involved in the orchestration of tissue-damaging immune response in states of chronic inflammation [2]. In recent years, the advent of sophisticated techniques of cellular biology has led to the identification of a new class of innate cells, termed innate lymphoid cells (ILCs), which have the ability to produce a vast array of cytokines mainly depending on their state of differentiation [3, 4]. ILCs play broad roles in lymphoid organogenesis, in the defense against extracellular pathogens, and in the maintenance of the epithelial barrier and are supposed to contribute to the amplification of immune-inflammatory responses in various organs [3, 4]. ILCs lack some cell lineage markers associated with T and B lymphocytes, dendritic cells, macrophages, and granulocytes but express CD90, CD25, and interleukin- (IL-) 7 receptor (R)*α* (CD127) (Table 1) [3, 4]. ILCs develop from hematopoietic precursors and their development is partially or wholly dependent on the common *γ*-chain, Notch, the transcription factor inhibitor of DNA binding-2 (Id2), IL-7, a cytokine involved in hematopoietic cell development and proliferation [3–6], and other transcription factors such as T-bet for ILC1, ROR*α* and Gata-3 for ILC2, and TCF1 and Gata-3 for ILC3 (Figure 1). Mice deficient for Id2 show largely normal development of T and B cells but lack all ILC subsets, suggesting the existence of a common Id2-expressing progenitor to ILC subsets [3–6].

ILCs are currently classified into three distinct populations on the basis of the expression of specific transcription factors and/or cell surface markers and their ability to secrete some profiles of effector cytokines. Thus, they closely resemble the heterogeneity of CD4^+^ T helper (Th) cell subsets. ILCs1 include classical NK cells and ILCs expressing T-bet, a Th-type-1-associated transcription factor, and producing interferon- (IFN-) *γ*, a potent stimulator of phagocyte activity against intracellular bacteria [3, 4, 7]. Conventional NK cells differ from ILCs1, because their development is dependent on the transcription factor eomesodermin (Eomes) and independent of Id2 [3, 4, 7, 8]. ILCs2 express the Th2-related transcription factor Gata-3 and, in humans, the chemokine receptor homologous molecule CRTH2 and produce IL-5, IL-9, IL-13, IL-4, and/or amphiregulin [3, 4] and play important roles in immunity to helminth infections [9–11] and in the pathogenesis of asthma and allergies [12–14]. ILCs3 express the transcription factor ROR*γ*t, synthesize IL-17A and IL-22, and, under specific stimuli, IFN-*γ*, and are involved in the recruitment of neutrophils, release of antimicrobial peptides, and epithelial cell proliferation (Figure 1) [3, 4]. Therefore, ILCs3 are required for the defense against bacterial infections [3, 4] and provide “help” to marginal zone B cells [15].

Here we review the available data on the role of ILCs in the control of both intestinal infections and effector cytokine response in immune-mediated pathologies of the gut.

## 2. ILCs1

At the present time, it is debated if typical ILCs1, which are predicted to be ROR*γ*t-independent, really exist in the gut, as IFN-*γ*-producing ILCs1-like cells described so far seem to originate from ILCs3 that upregulate T-bet and downregulate ROR*γ*t. NKp44-negative, c-kit-low ILCs expressing T-bet and IFN-*γ* and responding to IL-12, but not IL-23, with enhanced IFN-*γ* production are present in the intestinal lamina propria of patients with Crohn's disease (CD) but not in the fetal gut and in the noninflamed intestine of adults [16]. These cells are not seen in the intestinal lamina propria of alymphoid mice reconstituted with a human immune system under homeostatic conditions, but they appear following induction of colitis by dextran sodium sulphate (DSS) [16]. IFN-*γ*-producing, T-bet-positive, NKp44-negative, c-kit-low ILCs maintain, however, low levels of ROR*γ*t raising the possibility that they differentiate from ROR*γ*t-expressing ILCs3 in inflamed tissues. Fuchs and colleagues described an ILCs1-like subset, characterized by the expression of NKp44, NKp46, CD56, CD103, granzyme, and perforin and located in the intestinal epithelial compartment (intraepithelial ILC1-like cells). These cells respond to IL-12 and IL-15, but not IL-18, with enhanced secretion of IFN-*γ* [17]. Despite sharing the NKp44 marker with human ILCs3, intraepithelial ILC1 subset clearly differs from ILCs3 in terms of phenotype, function, and transcription factors involved in development. In particular, studies in mice demonstrated the requirement of NFIL3 and Tbx21 (the gene encoding T-bet) for their development, while RORc (the gene encoding ROR*γ*t) and aryl hydrocarbon receptor (AhR) were dispensable [17]. However, these cells differ also in various aspects from ILCs1, as they did not express IL-7R*α* and are independent of IL-15 for development and/or maintenance. Intraepithelial ILCs1 are increased in inflamed gut of CD patients and produce high amounts of IFN-*γ* in Rag1^−/−^ mice treated with anti-CD40, a model of colitis characterized by wasting syndrome and severe intestinal inflammation [17]. Depletion of such cells ameliorates colon inflammation, supporting their role in orchestrating detrimental responses in the gut [17].

Recently, a new common progenitor to all IL-7R-expressing ILC lineages, expressing Id2, has been identified and named CHILP. CHILP gives rise to peculiar ROR*γ*t-independent, T-bet-dependent, Eomes^−^, NKp46^+^, NK1.1^+^, IL-7R*α* ^+^ ILCs1. This cell type is present in the lamina propria of the small intestine and produces IFN-*γ* and TNF in response to IL-12 and to intestinal infection with the intracellular parasite* Toxoplasma gondii* [18]. Moreover, these cells express high levels of CXCR3, CXCR6, and CCR9, all of which are involved in lymphocyte migration to tissues. Studies in mice lacking specific genes revealed also that maintenance or differentiation of these cells requires T-bet, NFIL3, and Gata-3 as well as IL-15, but not IL-7 [18]. Another lymphoid precursor has been described in mouse fetal liver and adult bone marrow. It expresses high amounts of PLZF, a transcription factor previously associated with NK-T cell development, and has the potential to differentiate in ILC1, ILC2, and ILC3 [19].

## 3. Role of ILCs2 in the Control of Helminth Infections

Different types of Th2 cytokine-producing innate cells (e.g., natural helper cells, nuocytes, and type 2 innate helper cells), which express markers commonly found on ILC subsets (IL-7R*α*, c-kit, CD25, and CD90), have been described [5, 9, 10]. These cells, now collectively referred to as ILCs2, act downstream to IL-25 and IL-33 and make a substantial contribution to antihelminth immunity through their ability to produce IL-13, a cytokine that drives many of the physiological responses required for worm expulsion, such as mucus production and smooth muscle contractility [3, 4, 9, 10]. In this context it was shown that adoptive transfer of nuocytes into* Nippostrongylus brasiliensis*-infected IL17*β*R/IL1R1 mice (which are severely impaired in their ability to expel worms) enables these animals to efficiently eliminate the parasite and IL-13 secretion from nuocytes is essential for worm expulsion [9]. The ability of ILCs2 to combat parasites is dependent on T cells, because nuocytes fail to undergo sustained expansion in helminth-infected Rag2-deficient mice [9]. Similarly, adoptive transfer of natural helper cells into *γ*-chain-Rag2-deficient mice infected with* Nippostrongylus brasiliensis* resulted in the restoration of goblet-cell hyperplasia in the recipient mice [5]. Along the same line are the results obtained with adoptive transfer of type 2 innate helper cells into* Nippostrongylus brasiliensis*-infected *γ*-chain-Rag2-deficient mice, in which worm expulsion requires IL-25 administration [20].

ILCs2 also produce amphiregulin, an epithelial growth factor family member that is crucial for tissue repair following virus-induced inflammation [21] as well as enhancing the immunosuppressive properties of regulatory T cells during colitis [22]. ILCs2 express elevated levels of Gata-3, which is crucial for their development and maintenance [23]. Development of ILCs2 is also dependent on ROR*α* as mutant mice lacking this transcription factor do not have ILCs2 but display normal development of ILCs3 [24].

## 4. Regulation of ILCs3 Development and Activation

The factors/mechanisms that regulate differentiation/activation of ILCs3 are not fully understood, even though there is evidence that these cells can be activated by cytokines released by the intestinal epithelium and antigen presenting cells. While IL-7, stem cell factor, and TSLP are necessary for the development of ILCs3 and, together with IL-1, IL-2, and IL-15, regulate cell proliferation, IL-23 and IL-1*β* play an important role in inducing ILCs3 effector functions [25–30]. IL-23, a heterodimeric cytokine produced mainly by dendritic cells and macrophages, stimulates production of IL-22 by ILCs3 during intestinal infections [31, 32], but it is not essential for ILCs3 functions at steady state. Indeed, IL-23p19-deficient mice exhibit normal production of IL-22 by ILCs3 [33]. IL-23 induces also IL-17 production in CD56-negative ILCs3 isolated from the gut of patients with CD [34]. Another cytokine involved in the ILCs3 functions is IL-1*β*. IL-1*β* induces the accumulation and activation of ILCs3 during the course of* Helicobacter* (*H.*)* hepaticus* infection and synergizes with IL-23 or IL-7 in enhancing ILCs3-derived IL-22 production [35, 36]. Like CD4^+^Th17 cells, ILCs3 display a certain degree of context-dependent plasticity, as they are capable of acquiring functional characteristics of ILCs1. A subset of intestinal ILCs3, which is negative for CCR6 and either positive or negative for NKp46, coexpresses T-bet and ROR*γ*t [37, 38]. T-bet in ILCs3 controls expression of various target genes such as those encoding IFN-*γ*, Fas ligand, IL-12R*β*1, and CXCR3 [37, 38]. Interestingly, IL-22 production is evident in both T-bet^+^ and T-bet^−^ ILCs3, albeit at different levels, and T-bet is highly expressed in NKp46^+^ IL-22-producing ILCs3 in the intestine. Accordingly, mice deficient in Tbx21 have a reduced number of NKp46^+^ IL-22-producing ILCs3 in the intestinal lamina propria and failed to produce IFN-*γ* [37].

Collectively the available data indicate that CCR6-negative ILCs3 progress from a T-bet-negative to a T-bet-positive state and then acquire NKp46 expression. This later phenomenon can be followed by downregulation of ROR*γ*t [38, 39]. IL-7 is crucial not only for the development of ILCs but also for stabilizing in vivo the expression of ROR*γ*t in ILCs3, thus preventing their full conversion into IFN-*γ*-producing NKp46, T-bet-expressing ILCs [39].

The signals that induce T-bet expression in CCR6-negative ILCs3 are not completely understood. IL-12, the major Th1 inducing factor, seems to be uninvolved as mice deficient for IL-12 signaling have normal numbers of T-bet-expressing ILCs3 [38]. In contrast, IL-23-deficient mice have reduced numbers of T-bet-expressing ILCs3 [38], suggesting a role for IL-23 in inducing or maintaining T-bet expression in ILCs3. ILCs3 can also recognize and directly respond to environmental cues. For example, both mouse and human ILCs3 express AhR, a ligand inducible transcription factor that mediates a wide range of cellular events in response to halogenated aromatic hydrocarbons and nonhalogenated polycyclic aromatic hydrocarbons, small synthetic compounds, and metabolites of tryptophan and arachidonic acid. In its inactive state, AhR resides in the cytosol bound to several cochaperones. Following ligand binding, AhR dissociates from the chaperones and translocates to the nucleus, where it binds to its dimerization partner aryl hydrocarbon receptor nuclear translocator, and this complex activates the expression of a battery of genes with promoters containing a dioxin responsive element consensus sequence or a xenobiotic responsive element consensus sequence [40]. The number of postnatal ILCs3 as well as IL-22 but not IL-17 expression by ILCs3 in small intestine and colon is reduced in AhR-deficient mice [41]. The decrease in ILCs3 observed in AhR-deficient mice is not evident until the third week, suggesting that environmental stimuli may contribute to the differentiation, survival, and postnatal expansion of these cell subsets [41]. The pathways involved in the AhR-dependent ILCs3 development and function in the gut remain to be clarified, even though AhR ligands derived from food components could be involved. Indeed, it was shown that mice fed with phytochemical-free diets have a phenotype similar to AhR-deficient mice [42]. Another possibility is that AhR-dependent modulation of ILCs3 function is mediated by bacterial metabolites, as, under conditions of unrestricted tryptophan availability,* Lactobacilli* species produce indole-3-aldehyde, an AhR ligand, which enhances IL-22 expression in ILCs3 [43]. Interestingly, human ILCs3 express also RNA transcripts of Toll-like receptors (TLR) 1, 2, 5, 6, 7, and 9, though it seems that only TLR2 agonists induce cytokine production by human ILC3 in the presence of IL-2, IL-15, and IL-23 [44], supporting the hypothesis that bacteria can directly stimulate ILCs3 to synthesize effector cytokines.

Both human and mouse ILCs3 express NK cell activating receptors (e.g., NKG2D, DNAX accessory molecule-1, 2B4, CD94/NKG2C, NKp46, NKp44, and NKp30) that are known to mediate NK cell cytotoxicity and production of cytokines upon recognition of cognate cellular and viral ligands. In particular, NKp44 is detectable on ILCs3 and selectively marks the IL-22-producing subset in human tonsil and gut lamina propria [32, 36]. Engagement of NKp44 in ex vivo isolated ILCs3 selectively induces the expression of TNF and IL-2 while stimulation with IL-23, IL-1, and IL-7 preferentially induces IL-22 and GM-CSF expression [36]. Therefore, ILCs3, whose development is Notch dependent [45], can switch between IL-22 and TNF production, depending on the triggering stimulus.

## 5. The Role of ILCs3 in the Control of Intestinal Epithelial Barrier, Infections, and Inflammation

ILCs3 are involved in the development of intestinal lymphoid organs such as cryptopatches, which are located in the lamina propria between the gut crypts, and isolated lymphoid follicles, which represent important sites of T-cell-independent IgA production [3, 4]. For a detailed description of the regulatory functions of ILCs3 in the development of intestinal lymphoid organs, the reader is directed towards recent reviews [3, 4].

In healthy mammals, commensal bacteria are anatomically restricted either to the intestinal lumen, to the epithelial surface, or within the underlying gut-associated lymphoid tissues, a process that is essential to limit inflammation and maintain normal systemic immune homeostasis. ILCs3 have a critical role in this phenomenon, as their depletion in mice results in peripheral dissemination of commensal bacteria such as* Alcaligenes *species, residing within Peyer's patches and mesenteric lymph nodes of healthy humans and mice, and systemic inflammation [46]. ROR*γ*t-expressing ILCs3 express major histocompatibility complex class II (MHCII) and can process and present antigen to CD4^+^ T cells. Among ILCs3, MHCII is highly expressed on cells that lack T-bet and NKp46, while minimal expression occurs in cells positive for those markers. Moreover, MHCII is seen on ILCs2 but not ILCs1 [46]. Interestingly, ROR*γ*t-expressing, MHCII-positive ILCs3 lack expression of classical costimulatory molecules, such as CD40, CD80, and CD86, and therefore antigen presentation by these cells limits, rather than promoting, CD4^+^ T cell responses, through a mechanism that is independent of the ability of ILCs to produce IL-22 or IL-17A [33]. Mice lacking MHCII selectively on ILCs3 exhibit increased frequencies of proliferating CD4^+^ T cells in the blood, significant increase in commensal bacteria-specific serum IgG, and development of colitis characterized by enhanced production of IFN-*γ*, IL-17A, and TNF by mucosal CD4^+^ T cells [46]. All these phenomena can be prevented by administration of antibiotics, demonstrating a crucial role for commensal bacteria in the development of the disease [33, 46]. The ILC-mediated containment of commensal bacteria and regulation of mucosal homeostasis could also rely on the ability of ILCs3 to produce IL-22 in response to AhR-activating stimuli [41]. Indeed, binding of IL-22 to its heterodimeric receptor, comprising IL-10R2 and IL-22R1, on epithelial cells triggers the transcription factor STAT3, thereby promoting synthesis of antimicrobial peptides and proteins (i.e., *β*-defensins, RegIII*β* and RegIII*γ*, calgranulins S100A8 and S100A9, and lipocalin-2) and elevated levels of mucus-associated molecules (i.e., Muc1, Muc3, Muc10, and Muc13), with the downstream effect of limiting the translocation of commensal bacteria across the epithelial barrier during inflammation [47]. Although microbiota can modulate production of IL-22 by ILCs3 [33], the development of such cells seems to be independent of commensal bacteria, as the frequencies of IL-22-producing ILCs3 are similar in conventional versus germ-free mice [46].

ILCs3 also play an important role in the defense against pathogen infections, such as* Citrobacter rodentium*, a murine pathogen that models human enterohemorrhagic and enteropathogenic* Escherichia coli* infections [31, 32]. In particular, it was shown that mice lacking T and NK cells but retaining NKp46-expressing ILCs3 developed an IL-23-driven IL-22-mediated response and were resistant to infection [48]. ILCs3 also provide an early source of IL-22 during* Candida albicans* fungal infection [49]. IFN-*γ* produced by T-bet-dependent CCR6-ILCs3 has been shown to contribute to the response against* Salmonella typhimurium* infection in mouse [38].

Due to their ability to modulate epithelial cell functions as well as respond against commensal bacteria and pathogens, it is tempting to speculate that ILCs3 can participate in the complex regulation of IBD-related mucosal response, given that there is evidence that IL-22 is protective in murine models of IBD [27, 50]. On the other hand, as above specified, IL-17A and IFN-*γ* from NKp46-negative ILCs3 contribute to sustain inflammation in innate IBD models, such as anti-CD40 or* H. hepaticus*-induced colitis [25, 51]. These later findings are supported by the demonstration that IL-17A and IFN-*γ*-producing CD127^+^CD56^−^ILCs3 accumulate in inflamed gut of patients with CD [52]. However, another study documented a reduced frequency of NKp44^+^NKp46^−^ILCs type-3-like cells in CD [34]. Additional support to the hypothesis that ILCs3 can be proinflammatory in the gut comes from research into Tbx21/Rag2 double-knockout mice that develop spontaneous intestinal inflammation resembling UC [53]. In these mutants, ILCs3 produce high levels of IL-17A, a finding not observed in control mice, and depletion of all ILCs or neutralization of IL-17A improves colitis [54]. In addition, alymphoid mice on a Tbx21-deficient background do not develop colitis, indicating that lymphoid cells are required for inflammation [54].

A more complex scenario emerged however from studies in chronic CD45RB (high) CD4^+^ T cell transfer and anti-CD40 antibody-induced acute innate colitis models in Rag1-deficient mice showing that IL-23R signaling in ILCs3 is protective in the former and pathogenic in the latter [51]. Furthermore, it was shown that IL-23R signaling promotes innate colitis via IL-22 as neutralization of IL-22 protects mice from colitis and the adding back of IL-22 to IL-23R-deficient animals restores the disease [51].

## 6. Conclusions

In recent years, it became evident that ILCs play a fundamental role in immune responses, not only as first barrier against pathogens but also for their ability to influence downstream adaptive immune steps. These advances have been facilitated by the better characterization of the factors involved in the differentiation and maintenance of such cells as well as identification of the ILC subsets involved in specific immune responses. Environmental cues can promote the activation of ILCs as well as shifting from a subset to another one. In this context, it has been demonstrated that commensal microbiota-driven induction of IL-7 is determinant in the maintenance of ILCs3 and activation of AhR, an intracellular receptor for various environmental molecules, regulates the function of these cells in the gut. The intestine also contains additional stimuli, such as TLR ligands and cytokines, that could contribute to ILC development and activation. Much work remains however to be performed in order to ascertain the exact contribution that each ILC subset plays in the maintenance of immune homeostasis in the gut and how this task is accomplished. Further experimentation will be also needed to investigate whether and which ILCs are involved in the pathogenesis of chronic inflammatory diseases of the gut, such as CD, UC, and celiac disease, since cytokines produced by these cells are known to regulate the tissue-damaging immune responses in these disorders. At the same time, it would be relevant to investigate whether ILCs play a role in the process of colitis-associated colon cancer by either providing an immunosuppressive environment that eventually promotes tumor growth or amplifying cytotoxic pathways that kill tumor cells. In this context, it is noteworthy that IL-22 produced by ILCs facilitates the growth of cancer cells through a STAT3-dependent mechanism in a bacteria-driven mouse model of colorectal cancer [55].

## Figures and Tables

**Table 1:** Markers expressed by various subsets of innate lymphoid cells (ILCs).

ILC group	ILC lineage	Marker
1	ILC1	LIN−, CD25 low, CD56−, IL-7R*α*+, CD161+/−, NKp44−, NKp46−, IL-1R+, ICOS+, IL-12R*β*2+, and CRTH2−
	NK cells	LIN−, CD56+, CD25+/−, IL-7R*α*+/−, CD161+/−, NKp44+/−, NKp46+, IL-12R*β*2+, and CRTH2−

2	ILC2	LIN−, CD25 low, CD117+/−, IL-7R*α*+, ICOS+, IL-1R+, ST2+, IL-17RB+, CRTH2+, and CD161+

3	ILC3	LIN−, CD25 low, CD117+, IL-7R*α*+, CD161+, NKp44+, NKp46+, CRTH−, IL-1R+, IL-23+, and ICOS+
	LTi cells	LIN−, CD117+, IL-7R*α*+, CD161+/−, IL-1R+, and IL-23+

LIN, lineage; NK, natural killer; LTi, lymphoid tissue-inducer; IL, interleukin; CRTH2, chemoattractant receptor homologous molecule; ICOS, inducible T cell costimulator; R, receptor; ST2, subunit of IL-33R.
